# Protective Role of Black Tea Extract against Nonalcoholic Steatohepatitis-Induced Skeletal Dysfunction

**DOI:** 10.4061/2011/426863

**Published:** 2011-06-23

**Authors:** Subhra Karmakar, Sangita Majumdar, Anasuya Maiti, Monalisa Choudhury, Aniruddha Ghosh, Asankur S. Das, Chandan Mitra

**Affiliations:** ^1^Pre-Clinical Physiology Laboratory, Tripura Institute of Paramedical Sciences, Hapania, Tripura 799014, India; ^2^Department of Physiology, Presidency College, Kolkata, Kolkata, India; ^3^Institute of Genetic Medicine and Genomic Science, Madhyamgram, Kolkata 700 128, India

## Abstract

*Aim*. This paper aimed to examine the chemoprotective actions of aqueous black tea extract (BTE) against nonalcoholic steatohepatitis- (NASH-) induced skeletal changes in rats. *Material*. Wistar rats (body wt. 155–175 g) of both sexes, aged 4–5 months, were randomly assigned to 3 groups; Group A (control), Group B (60% high-fat diet; HFD), and Group C (HFD + 2.5% BTE). *Methods*. Several urinary (calcium, phosphate, creatinine, and calcium-to-creatinine ratio) serum (alkaline phosphatase and serum tartrate-resistant acid phosphatase), and molecular markers of bone turnover (receptor activator of NF-*κ*B ligand (RANKL), osteoprotegerin (OPG), and estrogen) were tested. Also, several bone parameters (bone density, bone tensile strength, bone mineral content, and bone histology) and calcium homeostasis were checked. *Results*. Results indicated that HFD-induced alterations in urinary, serum, and bone parameters as well as calcium homeostasis, all could be significantly ameliorated by BTE supplementation. *Conclusion*. Results suggest a potential role of BTE as a protective agent against NASH-induced changes in bone metabolism in rats.

## 1. Introduction

Nonalcoholic steatohepatitis (NASH) is a condition, which is described as accumulation of fat in liver with inflammation. This disorder may be observed in patients with no history of significant alcohol consumption, though histological examination may resemble alcohol-induced liver injury. It indeed may be the first clinical indication of insulin resistance, with complication of high blood pressure, coronary heart disease (CHD), and type 2 diabetes [1, 2]. It is claimed that, after cancer, cirrhosis from NASH is now the second most common age-related cause of death in type II diabetes [3]. Similar alarming observation is the association between human bone diseases with liver diseases, like viral liver cirrhosis is a risk factor for increased loss of minerals from bone [4, 5]. In an identical manner, patients with cholestatic liver disease have low bone turnover [6]. Even earlier [7, 8], it was reported that hepatic diseases are frequently associated with metabolic bone disorders. Hepatic osteodystrophy was reported to occur in up to 50% of patients with chronic liver disease (CLD). The results of this report suggested that increased bone resorption and less bone formation are the predominant cause of hepatic osteodystrophy in patients with CLD [9]. Osteopenia and osteoporosis are common complications of chronic liver disease. Patients with chronic liver disease may have other complications, such as malnutrition, loss of muscle mass, hypogonadism, low calcium intake, and vitamin D deficiency, all of which alter normal bone metabolism [10]. Several earlier studies had indicated the possible role of lipids in osteoporosis. High lipid levels were found closely related with decrease in bone mineral density (BMD) coupled with osteoporosis [11–13] as well as other disorders that may be a consequence of high lipids, including cardiovascular calcification [14–16] and atherosclerosis [11]. Consistent with these findings are the results of two in vivo studies that have shown that mice [17] and chickens [18] fed on a high-fat, high-cholesterol diet have reduced bone mineral density. An earlier study also had indicated that diets with high saturated fat content can produce deleterious effects on the absorption of dietary calcium and consequently an adverse effect on bone mineralization in growing animals [19]. Perhaps even more significant are data reporting that the loss in bone mineral density by high lipid diet can be revived with antioxidants, suggesting a possible mechanism of action through suppression of lipid oxidation levels [18].

Tea (*Camellia sinensis*) is rich in polyphenolic compounds, collectively known as the tea flavonoids. Its therapeutic value come forefront with numerous reports on tea as antioxidants, hypolipidemic, antineoplastic, and hypocholesterolemic [20–23]. Tea also has been reported to possess a cardiovascular-protective effect [24, 25]. In an epidemiological report it has been suggested that skeletal health is better preserved in aged women who drank tea than nontea drinkers [26]. Similar epidemiological studies have confirmed that tea drinking may be a possible preventive measure to reduce risk of bone loss in postmenopausal women as well as in men by increasing BMD [27, 28]. Estrogenic activity of naturally occurring isoflavones by virtue of their ability to bind nuclear estrogen receptor was reported earlier [29], and, recently, soy isoflavones with weak estrogen-like activities have been reported to modulate bone metabolism and serum lipids in perimenopausal women [30]. A recent study has further indicated that soy isoflavone can reduce the risk of obesity and preserve bone health in menopausal women [31]. Moderate consumption of tea, which contains flavonoids closely related to soy isoflavones, has been reported to be associated with higher BMD in men and women [26, 27] and lower rates of fracture [32, 33]. Results of series of studies from our laboratory with aqueous black tea extract [34–36] further support the idea that phytocompounds have efficacy in preventing bone loss. This study aimed to examine the protective role of black tea extract (BTE) against nonalcoholic steatohepatitis- (NASH-) induced skeletal dysfunction.

## 2. Methods

### 2.1. Animals

As NASH does not have any gender specificity and is seen in both sexes, animals from both sexes were selected for this study. Wistar rats, both male and female, aged 4-5 months, weighing 155–170 g were procured from a local authorized breeder registered under the Committee for the Purpose of Control and Supervision of Experiments on Animals (CPCSEA), Ministry of Environment and Forests, Government of India. Upon arrival at our institute, all animals were kept under standard conditions (12 hours light/dark schedule and at 25 ± 2°C room temperature throughout the experimental period) for a week with free access to drinking water and standard laboratory diet composed of 71% carbohydrate, 18% protein, 7% fat, and 4% salt mixture [37]. All animal experiments were performed according to the ethical guidelines suggested by the Institutional Animal Ethics Committee (IAEC) and Committee for the Purpose of Control and Supervision of Experiments on Animals (CPCSEA), Ministry of Environment and Forests, Government of India.

### 2.2. Diet and Treatment

Eighteen animals were randomly assigned to three groups: Group A (regular diet), Group B (high-fat diet), and Group C (high-fat diet + BTE). Each group contained six animals, three male and three female. Group A animals were fed standard laboratory diet [37] containing 71% carbohydrate, 18% protein, 7% fat, and 4% salt mixture. Group B animals were fed high-fat diet [38] containing carbohydrate: 22%, fat: 60% containing corn oil (palmitic acid: 6.3–18.2%, stearic acid: 0.9–4.5%, oleic acid: 18.5–46.1%, linoleic acid: 36.6–66.8%, linolenic acid: 0–2%, and arachidonic acid: 0–1.4%) [39], and protein: 18%. Animals of group C, in addition to high fat diet, were fed 2.5% (25 g/L) BTE by gavage technique for 30 days at a single dose of 10 mL/kg/day [40]. Animals of group A and B were given deionized water by gavage technique, 10 mL/kg/day as vehicle. In our preliminary studies, a fourth group (Group D) of animals was included containing six animals (male 3, female 3), which were given standard laboratory diet simultaneously with 2.5% BTE by gavage technique for 30 days at a single dose of 10 mL/kg/day. As aqueous black tea extract in Group D animals independently could not produce any significant variation of results in marker parameters of NASH, compared to control, this group was not considered for further detailed study to restrict animal use as per recommendation of the Animal Ethics Committee.

### 2.3. Preparation of Aqueous BTE

The black tea (*Camellia sinensis*) extract was prepared from CTC (Curl, Tear and Crush) BOP- (Broken Orange Pickoe-) grade black clonal tea. It was processed and supplied by Tocklai Experimental Station, Jorhat, Assam, India to the Drug Development Division, Indian Institute of Chemical Biology, Jadavpur, Kolkata, India. We received a generous gift from that institute. A fresh 2.5% aqueous BTE was prepared everyday following the method of Wei et al. [40]. Twenty-five gram of black tea was added to 500 mL of boiling water and was steeped for 15 min. The infusion was cooled to room temperature and then filtered. The tea leaves were extracted a second time with 500 mL of boiling water and filtered, and the two filtrates were combined to obtain a 2.5% aqueous black tea extract (2.5 g of tea leaf/100 mL water). The resulting clear solution is similar to tea brews consumed by humans.

### 2.4. Selection of Effective Dose of High Fat Diet and Black Tea Extract

For determination of effective dose (ED) of high fat diet (HFD) and black tea extract (BTE), dose response studies were undertaken. In case of high fat, it was observed that although significant changes could be seen from 30% of high fat onward, but both biochemical and histological changes were most prominent with 60% HFD. Similar changes with 60% HFD were reported earlier [38]. As for BTE, a submaximal dose response study revealed that significant recovery responses were seen with doses between 2-3% (w/v) aqueous BTE. Recovery responses with similar dose of BTE were also reported earlier [40].

### 2.5. Urine Collection

Fasting urine was collected for 24 hours (9 AM to next day 9 AM) according to the standard laboratory procedure [41] as described elsewhere by Chanda et al. [37]. Care was taken so that no urine was lost through evaporation. Total volume of urine was measured, and the following parameters were assessed.

### 2.6. Estimation of Urinary Parameters

#### 2.6.1. Urinary Calcium

Urinary calcium was measured according to the method of Adeniyi et al. [42]. The assay of calcium was based on the principle that metal complexing dye orthocresopthalein complexone (CPC) forms a chromophore with calcium in alkaline solution. Diethylamine (DEA) were added to enhance the colour intensity. For urinary calcium estimation, to 20 *μ*L of urine 1 mL CPC reagent and 1 mL DEA buffer was added. Colour intensity was measured at 575 nm against a blank by using a UV-double beam spectrophotometer (Shimadzu 160 A; Shimadzu Corporation, Kyoto, Japan). Calcium carbonate (CaCO_3_) solution pH-3.0 was used as standard. All the reagents were prepared with calcium-free triple distilled water.

#### 2.6.2. Urinary Phosphate

Urinary phosphate was measured according to the method of Lowry and Lopez [43]. For urinary phosphate estimation, 10 *μ*L of urine was diluted 50 times with distilled water and to it 1 mL molybdate reagent and 200 *μ*L amino naphthol sulfonic acid (ANSA) was added, mixed well, and kept in dark for 10 minutes. Reading was taken at 630 nm against blank. The concentration of phosphate in urine was calculated using a standard curve of monopotassium phosphate (KH_2_PO_4_).

#### 2.6.3. Urinary Creatinine

Urinary creatinine was measured according to the method of R. L. Nath and R. K. Nath [44]. For urinary creatinine estimation, 50 *μ*L of urine was diluted 10 times and to it 2 mL saturated picric acid and 150 *μ*L 10% sodium hydroxide solution were added, mixed and kept for 15 minutes at 37°C incubator. To it, 5 mL of water was added. Reading was taken at 520 nm against blank. The concentration of creatinine in urine was calculated using a standard curve of creatinine.

### 2.7. Serum Collection

Blood was collected directly from the heart under urethane anesthesia (1.7 mg/g body weight). Serum was obtained by using standard laboratory protocol.

### 2.8. Estimation of Serum Alkaline Phosphatase (AP) Activity

Serum alkaline phosphatase was measured using p-nitrophenyl phosphate as substrate [45]. Alkaline phosphatase activity was measured by the hydrolysis of p-NPP (paranitrophenyl phosphate) at pH-10.8 using glycine-NaOH buffer at 37°C. In brief, 0.5 mL of 5.5 mM p-NPP in 0.4 mL of glycine-NaOH buffer (pH-10.8) solution were pipetted in a test tube and incubated at 37°C for 10 minutes. After incubation, 0.05 mL of sample was added, mixed, and incubated at 37°C for 30 minutes. Reaction was stopped by adding 4 mL of 0.1 N NaOH, and reading was taken at 410 nm against a blank in a spectrophotometer (Shimadzu 160 A; Shimadzu Corporation, Kyoto, Japan).

### 2.9. Estimation of Serum Tartrate-Resistant Acid Phosphatase (TRAP) Activity

Serum TRAP activity was estimated by kinetic method by using reagent kit (LABKIT, Spain). Briefly, unhemolyzed serum was mixed with the test reagent (10 mM *α*-naphthyl phosphate, 6 mM Fast Red TR, and 2 mM sodium tartrate in 50 mM sodium citrate buffer, pH-5.2) and the absorbance of the sample was read at 405 nm at 1 minute interval thereafter for 3 minutes. The difference of absorbance and the average absorbance difference per minute (ΔA/min) was calculated [46].

### 2.10. Estimation of Serum Estradiol

Serum was obtained by using standard laboratory protocol. Serum estrogen level (pg/mL) was determined by using the ELISA EIAgen Estradiol kit (Adaltis Italia, Italy). All samples were assayed in duplicate. The intra-assay coefficient of variation was 9.08%. To avoid inter-assay variation all samples were run at one time.

### 2.11. Estimation of Serum RANKL

Serum RANKL was estimated by using the ELISA kits (Quantikine, R & D Systems Inc., Minneapolis, MN, USA). All samples were assayed in duplicate. The intra-assay variation was 11.75%. All samples were run at one time to avoid inter-assay variation. Optical density of each well was determined by using a microplate reader (Thermo Labsystems, Finland).

### 2.12. Estimation of Serum Osteoprotegerin (OPG)

Serum OPG was estimated by using the ELISA kits (Quantikine, R & D Systems Inc., Minneapolis, MN, USA). All samples were assayed in duplicate. The intra-assay variation was 10.09%. All samples were run at one time to avoid inter-assay variation. Optical density of each well was determined by using a microplate reader (Thermo Labsystems, Finland).

### 2.13. Measurement of Bone Density

The right femur, eighth thoracic rib, eighth thoracic vertebra, and fourth lumbar vertebra were freed off soft tissue and cleaned using small scissors, tweezers, and cotton gauze. Before measurement of the density, femur was cut at the middiaphysis and the marrow was washed out. Bone density was measured according to the method described by Arjmandi et al. [47] by using Archimedes' principle. Briefly, each bone was put in an unstoppered vial filled with deionized water, and the vial was placed under a vacuum for 90 minutes to ensure that all the trapped air diffused out of the bone. Each bone was removed from the vial, blotted with gauze sponge, weighed, and returned to the vial containing deionized water. The bone was reweighed in water and density was calculated (g/cm^3^ bone volume).

### 2.14. Estimation of Bone Tensile Strength

After sacrifice of the animal, left femur was excised and cleaned off adhering soft tissues. Bone tensile strength was measured as described by Shapiro and Heaney [48] using a hand-held force meter (Excel Enterprises, India). Briefly, the femur was supported latitudinally on each end, and pressure was placed directly onto the middle of the bone until it fractured. The breaking force (kg) was recorded.

### 2.15. Estimation of Bone Calcium and Bone Phosphate Level

Right femur, eighth thoracic rib, eighth thoracic vertebra, and fourth lumbar vertebra were removed and cleaned of adhering tissue. The whole bone was extracted two times with a 1 : 1 mixture of ethanol and diethyl ether for 48 hours and one time with diethyl ether for 24 hours. The dehydrated and defatted bones were turned into ash at 600˚C for 48 hours and hydrolyzed in 6 N HCl for determination of calcium and phosphate [49]. Calcium and phosphate were estimated according to the method as described, respectively, by Adeniyi et al. [42] and Lowry and Lopez [43].

### 2.16. Preparation of Intestinal Loops

After the experimental period was over, the animals of all groups were fasted for 16 hours. The preparation of animals and intestinal loops for the study of calcium transference in situ was made by following the method as described elsewhere by Islam et al. [50]. Briefly, the animal was anesthetized (urethane, 1.7 mg/kg body weight), the abdomen of each animal was opened through a midline longitudinal incision, the bile duct was ligated and duodenal, jejunal, and ileal segments were located. Two ligatures, one proximal and the other distal, were applied tightly in each loop measuring about 8 cm in all duodenal, jejunal, and ileal segments. Loops were so selected that each contained 8–10 vessels, and care was taken so that no major blood vessels were occluded by the ligature.

### 2.17. Measurement of Intestinal Calcium Transference

For the measurement of intestinal calcium transference, 1 mL of prewarmed (37°C) Tris-HCl buffer solution containing 0.2 mM CaCl_2_ was injected with a 25-gauge needle in each ligated segment. The intestinal loops were placed in their usual position and the abdomen was closed. After one hour, animals were sacrificed, the preselected loops were removed and the fluid from each loop was collected separately, together with a few washings of the lumen with triple distilled water. The collected fluid was then increased to a definite volume with calcium free triple distilled water. A fraction of this fluid was then used for the estimation of Ca^2+^ using a double beam spectrophotometer (Shimadzu, UV-160 A) according to the method described by Adeniyi et al. [42]. The difference between the amount of Ca^2+^ introduced and the amount left unabsorbed was used to estimate the amount of Ca^2+^ absorbed. The intestinal part constituting the loop was dried on a watch glass in an electric oven at 90°C to attain a constant weight, which was recorded as the weight of the dried loop. Intestinal transference of calcium was expressed as *μ*M of calcium/g of dry weight/hour.

### 2.18. Preparation of Intestinal Mucosal Extract

After sacrificing the animal and opening of the abdomen, the whole of the small intestine was quickly removed. The portion comprised of the duodenum, jejunum, and ileum were separated and chilled in ice. Intestinal mucosa was collected as described by Maenz and Cheeseman [51], and the scrapings were homogenized according to the method of Koyama et al. [52].

### 2.19. Estimation of Intestinal Mucosal Alkaline Phosphatase Activity

The activity of alkaline phosphatase of intestinal mucosa was estimated by using p-nitrophenyl phosphate as substrate [45]. The protein content of the homogenate used for the study was determined using the method of Lowry et al. [53].

### 2.20. Estimation of Intestinal Mucosal Ca^2+^ Activated ATPase Activity

The activity of the enzyme Ca^2+^-ATPase was also studied from the mucosal extract using the method of Rorive and Kleinzellar [54]. The assay was based on the principle that the release of inorganic phosphate (Pi) from ATP is measured in presence of either Ca^2+^ or Mg^2+^. For the assay of mucosal Ca^2+^, activated ATPase, 250 *μ*L 0.4 M Tris-HCl buffer pH-7.4 and 250 *μ*L 40 mM ATP (Tris-salt, Sigma) were added into three of the test tubes placed on ice. To tubes 1 and 3, 250 *μ*L of 40 mM CaCl_2_ were added. At time zero, the reaction was started by addition of 250 *μ*L of mucosal homogenate to tubes 1 and 2. The volumes in all tubes were adjusted to 2 mL with calcium free distilled water. The three tubes were then incubated at 37°C with gentle shaking for 30 minutes. Under this condition the release of Pi is linear unto 60 mins. The reaction was stopped by transfer of the tubes to ice and addition of 0.4 mL ice-cold 35% (w/v) trichloro acetic acid (TCA). The tubes were then centrifuged for 10 minutes at 10,000 rpm in a refrigerated centrifuge. The protein pellet was dissolved in 1 mL of 1 N sodium hydroxide (NaOH), and protein content was determined following the method of Lowry et al. [53]. Phosphate liberated during Ca^2+^ ATPase enzyme activity was estimated by the method of Lowry and Lopez [43].

### 2.21. Bone Tissue Collection and Processing

The left proximal tibia and fourth lumbar vertebra were removed, dissected free of soft tissue, and fixed with 4% paraformaldehyde for 16–18 hours at 4°C. After fixation, specimens were washed for 12 hours at 5°C in each of the following series of solutions: 0.01 M PBS containing 5% glycerol, 0.01 M PBS containing 10% glycerol, and 0.01 M PBS containing 15% glycerol. The specimens were then decalcified in EDTA-G solution (14.5 g EDTA, 1.25 g NaOH, and 15 mL glycerol, pH-7.3) for 10–14 days. The decalcified tissues were washed sequentially at 5°C for 12 hours in (a) 15% sucrose and 15% glycerol in PBS, (b) 20% sucrose and 10% glycerol in PBS, (c) 20% sucrose and 5% glycerol in PBS, (d) 20% sucrose in PBS; 10% sucrose in PBS, (e) 5% sucrose in PBS, and (f) 100% PBS. Then the tissues were washed with PBS and dehydrated in graded series of alcohols, followed by clearing in xylene, and finally embedded in paraffin [55]. The specimen were cut into 5-6 *μ*m sections and stained with haematoxylin-eosin. Representative sections were observed and photomicrography was performed with the help of a bright field microscope equipped with a digital camera (Carl Zeiss, Germany).

### 2.22. Statistical Analysis

Data were expressed as mean ± S.E.M. obtained from a particular group comprising both male and female animals. Data were compared using Kruskal-Wallis nonparametric ANOVA followed by Mann-Whitney “U” multiple comparison test (software version 2.6.5, StatsDirect, UK). Differences were considered significant if *P* < .05.

## 3. Results

### 3.1. Urinary Calcium, Phosphate, and Creatinine Excretion Profiles and Calcium-to-Creatinine Ratio

The urinary calcium, phosphate, and creatinine excretion profiles together with Ca : Cr ratio of control group (Group A), HFD (Group B), and HFD + BTE (Group C) are shown in Table 1. Compared to control group, animals of HFD-fed group showed a significant increase in all the urinary parameters studied, namely, calcium, phosphate, creatinine, and Ca : Cr ratio (*P* < .01). Elevated response of all these parameters was significantly counter regulated in the HFD + BTE-supplemented group of rats (Group C).

### 3.2. Serum Alkaline Phosphatase (AP) and Tartrate-Resistant Acid Phosphatase (TRAP) Activity Profiles

The serum alkaline phosphatase (AP) activity profile of rats of control, HFD, and HFD + BTE supplemented groups are shown in Table 2. Rats of HFD-fed group (Group B) showed a significant increase in serum alkaline phosphatase activity when compared to animals of control group (*P* < .01) (Group A). This increase in AP activity was significantly lowered (*P* < .01) in rats receiving BTE (Group C). Likewise, the significant increase (*P* < .01) in TRAP activity in HFD-fed group (Group B), compared to control (Group A), could be effectively reduced by aqueous BTE supplementation (Group C; Table 2).

### 3.3. Bone Density Profiles

Animals in the HFD-fed group (Group B) had significantly lower densities of the right femur (*P* < .01), eighth thoracic rib (*P* < .01), eighth thoracic vertebra (*P* < .01), and fourth lumbar vertebra (*P* < .01), compared with the control group (Group A). BTE supplementation could produce significant increase (*P* < .01) in bone density of all bones: right femur (*P* < .01), eighth thoracic rib (*P* < .01), eighth thoracic vertebra (*P* < .05), and fourth lumbar vertebra (*P* < .01; Table 3).

### 3.4. Bone Tensile Strength

**Figure 1:**
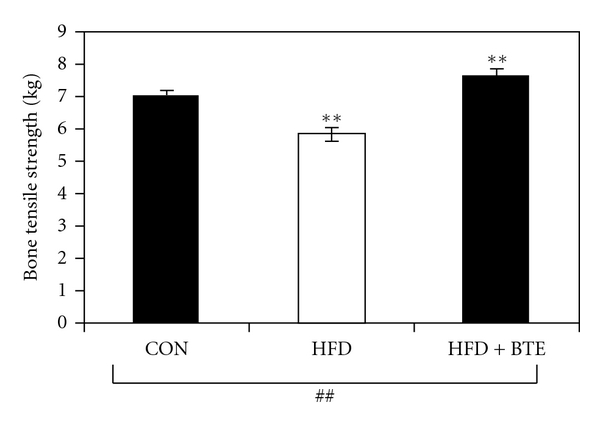
Graphical presentation of bone tensile strength of control (CON; Group A), HFD (Group B), and HFD + BTE supplemented (Group C) groups of animals. Values are expressed as mean ± S.E.M. (*n* = 6). ^##^ *P* < .01 denotes significance based on Kruskal-Wallis non-parametric ANOVA test, **P < .01 denotes significance based on Mann-Whitney “U” multiple comparison test.

Figure 1 shows that compared to control, bone tensile strength in HFD-fed animals was significantly reduced (*P* < .01). BTE supplementation in these animals was found effective in recovering (*P* < .01) this tensile strength back to control group level.

### 3.5. Bone Calcium and Bone Phosphate Levels

Results of bone calcium and phosphate levels are shown in Table 4. Animals of HFD-fed group (Group B), compared to control group (Group A), showed a marked decrease in calcium and phosphate levels of right femur (calcium: *P* < .01; phosphate: *P* < .01), eighth thoracic rib (calcium: *P* < .01; phosphate: *P* < .01), eighth thoracic vertebra (calcium: *P* < .01; phosphate: *P* < .01), and fourth lumbar vertebra (calcium: *P* < .01; phosphate: *P* < .01). When these HFD-fed animals were supplemented with BTE (Group C), significant recovery in content of both minerals was seen.

### 3.6. Serum Estradiol Level

**Figure 2:**
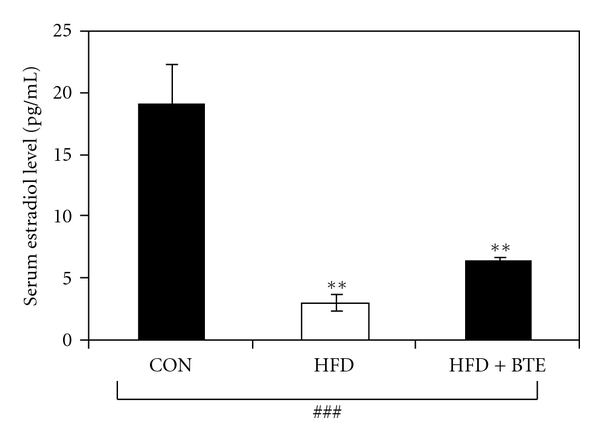
Serum estradiol level of control (CON; Group A), HFD treated (Group B), and HFD + BTE (Group C) supplemented female groups of rats. Values are expressed as mean ± S.E.M. (*n* = 6, female data only). ^###^ *P* < .001 denotes significance level based on Kruskal-Wallis nonparametric ANOVA test and **P < .01 denotes significance level based on Mann-Whitney “U” multiple comparison test.

Figure 2 shows the effects of supplementation of BTE on serum estradiol level of HFD, fed rats. Compared to control (Group A), a significant decrease in estradiol level was seen in HFD-fed (Group B) animals (*P* < .01). This could be recovered significantly (*P* < .01) when BTE was supplemented (Group C).

### 3.7. Serum RANKL and OPG Activity

**Figure 3:**
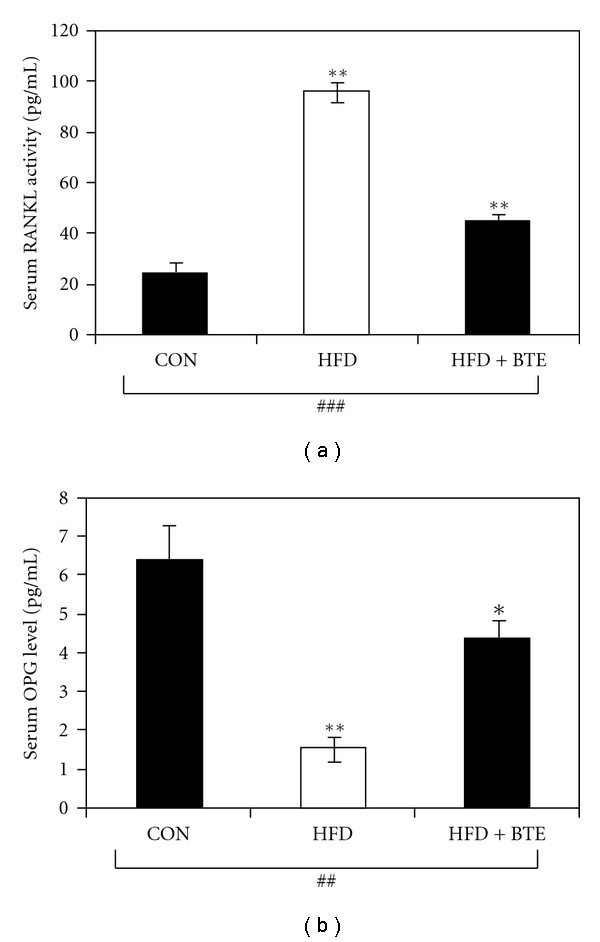
(a) Serum RANKL activity and (b) OPG level of control (CON; Group A), HFD treated (Group B), and HFD + BTE supplemented (Group C) groups of rats. Values are expressed as mean ± S.E.M. (*n* = 6). ^###^ *P* < .001 denotes significance level based on Kruskal-Wallis nonparametric ANOVA test, ^##^ *P* < .01 denotes significance level based on Kruskal-Wallis nonparametric ANOVA test, **P < .001 denotes significance level based on Mann-Whitney “U” multiple comparison test, and *P < .05 denotes significance level based on Mann-Whitney “U” multiple comparison test.

Figure 3 depicts serum levels of RANKL (Figure 3(a)) and OPG (Figure 3(b)) of different groups of rats. Compared to control, HFD-fed rats showed a decrease in serum OPG (*P* < .01) level, which was found significantly (*P* < .05) counter regulated on BTE supplementation. In contrast, compared to control, serum RANKL level was increased significantly (*P* < .01) in HFD-fed animals. On BTE supplementation, a significant decrease (*P* < .01) in the RANKL level was noted.

### 3.8. Intestinal Transference of Calcium

**Figure 4:**
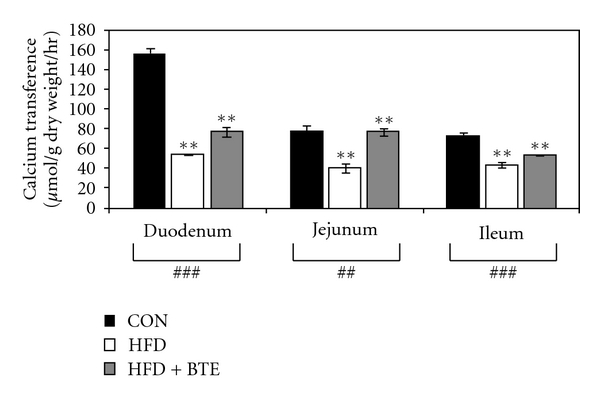
Transference of calcium in duodenal, jejunal, and ileal segments of control (CON; Group A), HFD treated (Group B), and HFD + BTE supplemented (Group C) groups of rats. Values are expressed as mean ± S.E.M. (*n* = 6). ^###^ and ^##^ denote significance level *P* < .001 and *P* < .01, respectively, based on Kruskal-Wallis nonparametric ANOVA test and **P < .01 denotes significance level based on Mann-Whitney “U” multiple comparison test.

Results in Figure 4 depicts that, compared to control, HFD-fed rats (Group B) showed a segment-wise reduction in intestinal transference of calcium (*P* < .01 for duodenum, jejunum, and ileum). BTE supplementation could significantly correct such alterations in calcium transference in HFD-fed rats (*P* < .01 for duodenum, jejunum, and ileum).

### 3.9. Calcium ATPase Activity

**Figure 5:**
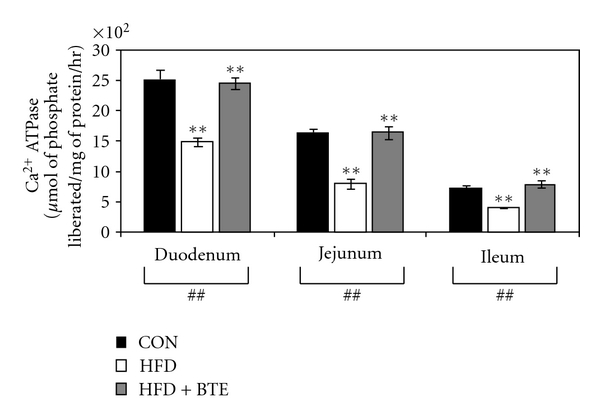
Ca^2+^-ATPase activity of intestinal mucosal extracts in duodenal, jejunal, and ileal segments of control (CON; Group A), HFD treated (Group B), and HFD + BTE supplemented (Group C) groups of rats. Values are expressed as mean ± S.E.M. (*n* = 6). ^##^ *P* < .01 denotes significance level based on Kruskal-Wallis nonparametric ANOVA test and **P < .01 denotes significance level based on Mann-Whitney “U” multiple comparison test.

Figure 5 shows the effects of feeding of HFD on the mucosal calcium ATPase activity of different segments of small intestine of rats. Results show that, compared to control rats, HFD feeding caused a significant reduction in the activity of this enzyme (*P* < .01 for duodenum, jejunum, and ileum), while BTE supplementation could significantly increase this enzyme activity (*P* < .01 for duodenum, jejunum, and ileum).

### 3.10. Intestinal Alkaline Phosphatase Activity

**Figure 6:**
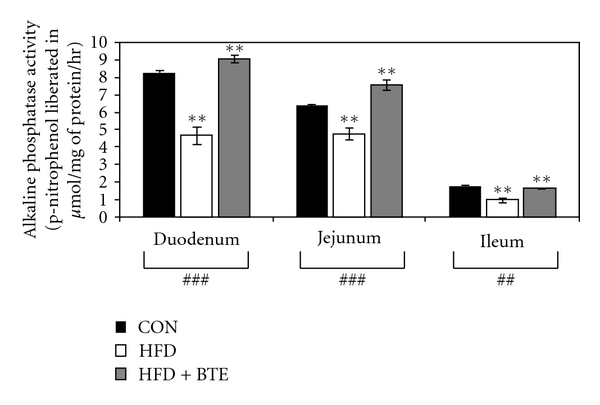
Alkaline phosphatase activity of intestinal mucosal extracts in duodenal, jejunal, and ileal segments of control (CON; Group A), HFD treated (Group B), and HFD + BTE supplemented (Group C) groups of rats. Values are expressed as mean ± S.E.M. (*n* = 6). ^###^ and ^##^ denote significance level *P* < .001 and *P* < .01, respectively, based on Kruskal-Wallis nonparametric ANOVA test and **P < .01 denotes significance level based on Mann-Whitney “U” multiple comparison test.

Figure 6 depicts that, compared to control group, HFD-fed rats showed a significant reduction in the activity of alkaline phosphatase in all segments of small intestine (*P* < .01 for duodenum, jejunum, and ileum). BTE supplementation was found significantly effective in restoring this enzyme activity in all these segments (*P* < .01 for duodenum, jejunum and ileum).

### 3.11. Histological Analysis of Cancellous and Cortical Bones

**Figure 7:**
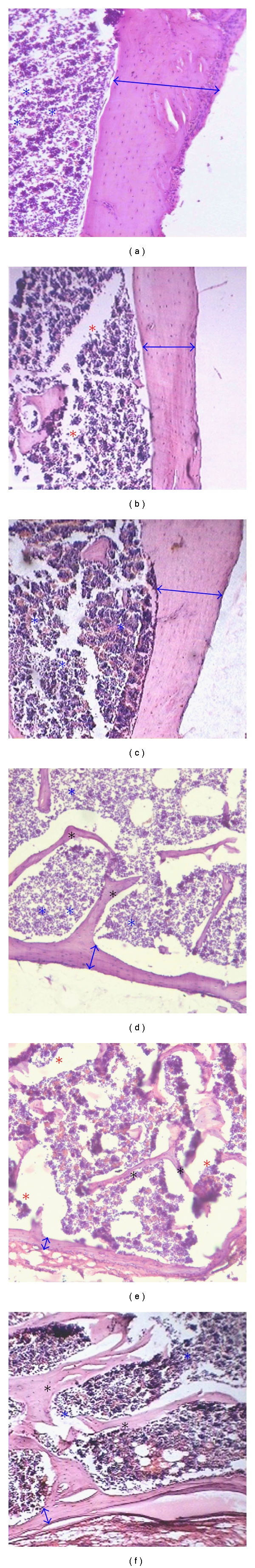
Histological analysis of tibia ((a)–(c)) and lumbar vertebra ((d)–(f)) of rats (hematoxylin-eosin staining, X 100). Representative photomicrograph from HFD group (b) with reduction in cortical thickness and empty bone marrow of the tibia than CON (a) and HFD + BTE (c). Photomicrograph of lumbar vertebra of HFD group (e) with reduction in cortical thickness, empty bone marrow and less trabecular bone compared with CON (d) and HFD + BTE group (f). Blue line, cortical thickness; blue star, bone marrow; red star, empty bone marrow; black star, trabecular thickness.

Histological studies of two different types of bones were conducted to determine the *in vivo* effects of BTE in the HFD-fed animals. HFD-fed rats (Group B) showed a decrease in cortical thickness and empty bone marrow space at the proximal tibia (Figures 7(a)–7(c)) and fourth lumbar vertebra (Figures 7(d)–7(f)), as compared to control (Group A). Additionally, there was a decrease in trabecular bone volume and thickness at the fourth lumbar vertebra. However, on BTE supplementation, bone repairing actions were seen in these HFD-fed animals.

## 4. Discussion

The present study examined the chemoprotective actions of aqueous black tea extract (BTE) against HFD fed (60%) NASH skeletal changes. Bone manifestations are well-known as extrahepatic complications of chronic liver disease. Patients with chronic liver disease are at increased risk of developing hepatic osteodystrophy manifested as osteomalacia or osteoporosis [56]. Osteoporosis is a frequent complication of end-stage liver disease irrespective of its etiology [57].

Recent research also suggests that there is a strong positive correlation between osteoporosis and hyperlipidemia. Excess lipid consumption may lead to lipid accumulation and subsequent oxidation within bone vasculature, wherein osteoblastic differentiation may be altered. In addition to their inhibitory role in osteoblastic differentiation, it is believed that because oxidized lipids induce endothelial expression of monocyte chemotactic factors, as well as other potent inducers of osteoclastic differentiation, oxidized lipids may also promote bone resorption [58].

Biochemical markers of bone turnover have been widely used as research tool to measure the effects of drugs on bone remodeling [59]. Elevated levels of bone markers are associated with rapid bone loss and may predict a greater risk of fracture independently of bone mineral density (BMD) variability [10]. In our study, compared to control, HFD treated rats showed an increase in urinary loss of calcium, phosphate, and creatinine (Table 1), which could be significantly corrected by BTE supplementation, suggesting BTE possibly has some positive role in preventing bone resorption and/or increase bone formation or both.

Results of studies of two other specific markers of bone turnover, namely, urinary calcium-to-creatinine (Ca : Cr) ratio and serum alkaline phosphatase (AP) further support our speculation (Tables 1 and 2, resp.). It was observed that BTE was effective in reducing HFD-induced increase in serum AP and urinary Ca : Cr ratio. Since a rise in AP and Ca : Cr ratio has been linked with collagen degradation, bone resorption and osteoporosis [60–63], it appeared that BTE was possibly effective in preventing bone loss. A close association of increased serum concentrations of TRAP as a potential index for osteoclastic activity is well established [64]. In the present study, compared to control, HFD-fed animals showed an increase in serum TRAP level (Table 2). However, this response was found well regulated by BTE supplementation, indicating that BTE possibly was effective even in controlling osteoclastic activity to preserve skeletal health.

Such speculations were cross-examined in our studies by measuring parameters like bone density (Table 3) and bone mineral content (Table 4). Rats in the HFD fed-group had significantly lower bone densities and mineral content, compared to control rats, which could be significantly regained by BTE supplementation, suggesting again the protective role of BTE against HFD-fed skeletal dysfunctional changes. Further confirmatory evidence in favor of our speculation was obtained in our experiment for bone tensile strength or breaking force, which is required to break the bone. Compared to control, HFD-fed animals showed lower tensile strength (Figure 1), which could be significantly elevated (increase in breaking force) on BTE supplementation. 

Chronic liver disease accelerates the development of hypogonadism due to both reduced hypothalamic release of gonadotrophins and primary gonadal failure. A decline in circulating estrogen may be a mediator of bone loss in women and men with chronic liver disease [10]. In the present study, HFD-fed female rats showed a significant decrease in serum estradiol level (Figure 2), which could be considerably restored by BTE supplementation. This result showed that BTE has efficacy as a potent phytoestrogenic compound. The estrogen-enhancing property of flavonoids [65, 66] from various extragonadal sites like mesenchymal cells of adipose tissue, osteoblasts, and chondriocytes of bone, numerous sites in the brain, breast, and vasculature [67, 68] possibly was responsible for such an increase in serum estradiol level by modulating the aromatase activity [68].

Osteoprotegerin (OPG) is a novel receptor that blocks osteoclast formation. Either in a cell membrane-bound or in a soluble form, RANKL, receptor activator of NF-kappa *β* (RANK), stimulates osteoclastogenesis and osteoclastic bone resorption. Factors that stimulate bone resorption increase RANKL expression and, with some exceptions, decrease OPG expression [69]. In the present study, animals of HFD-fed group showed a significant decrease in serum level of OPG (Figure 3(b)) and a significant increase in serum level of RANKL (Figure 3(a)), compared to control group of rats. BTE supplementation, however, could significantly restore altered levels of serum OPG and RANKL, suggesting a more confirmatory protective role of BTE against osteoclastic differentiation and activity in HFD-fed rats.

Calcium absorption has been reported to occur in body throughout the length of the small intestine, the absorption being greater in the duodenum and proximal jejunum than in the ileum [70]. The involvement of two intestinal enzymes, alkaline phosphatase, and Ca^2+^-ATPase, has been proposed frequently in this phenomenon because the activity of these enzymes correlates with the degree of calcium absorption in different parts of intestinal tract under different circumstances [50, 71, 72]. Results also indicate that Ca^2+^-induced ATP hydrolysis in the intestine is the result of two enzymatic activities, namely, alkaline phosphatase present in brush border as well as basolateral membranes and a more specific Ca^2+^-ATPase exclusively located in basolateral plasma membrane [73]. Evidence further indicates that estrogen is more directly involved in determining intestinal calcium absorption because decreased calcium absorption due to ovarian hormone insufficiency is corrected by hormone replacement therapy [74, 75]. In the present study, intestinal transference of Ca^2+^ was reduced in HFD-fed rats. Result indicated that deficiency of estrogen had a negative influence upon intestinal transference of calcium, as these animals showed a greater degree of decrease in the transference of calcium than the control animals (Figure 4). Such observation finds its support from earlier findings that estrogen may have direct role via the estrogen receptors in regulating intestinal calcium absorption *in vitro* and *in vivo* [76, 77]. BTE supplementation in the present study was found effective in correcting such reduction in calcium transference, indicating that BTE also possibly had positive influence upon mucosal transference of calcium.

To ascertain the mechanism of such decrease in intestinal transference of calcium, the activities of two most relevant mucosal calcium transferring enzymes, namely, alkaline phosphatase and calcium-ATPase were examined. Activities of both of these enzymes were found inhibited in HFD-fed group of animals (Figures 5 and 6). This supports well the earlier observations that both the enzymes are involved in calcium absorption as the activities of these enzymes correlate with the degree of calcium absorption in different parts of the intestinal tract under different circumstances [50, 71, 72]. BTE supplementation could well restore the activities of both of these enzymes indicating that the observed positive influence of BTE upon intestinal transference of calcium is thus mediated through modulation of activities of these transferring enzymes. 

Histological (Figure 7) observation of serial section of tibia and lumbar vertebrae revealed that, compared to control, significant reduction in cortical thickness and bone marrow content was seen in HFD fed rats. On BTE administration, these changes were significantly corrected, suggesting a potential bone repairing action of BTE.

It is recognized that animal studies contain some inherent design limitations. First, restrictions of animal use and therefore sample size; second, the doses required for demonstrating the disease prevention effects are usually higher than the amounts consumed by humans. Furthermore, because of infrastructure limitations it was necessary to rely on biochemical and histological measures rather than using peer reviewed DEXA-derived indices for examining bone profiles. To minimize the error associated with such limitations, we took extreme care while selecting the parameters, collecting data and analyzing them by well-recognized statistical methods followed by us [34, 35] earlier.

## 5. Conclusions

In conclusion, results of this study strongly suggest a protective role of BTE against NASH-induced changes in bone metabolism in rats.

## Figures and Tables

**Table 1:** Urinary excretion of calcium, phosphate, creatinine, and calcium : creatinine (Ca : Cr) ratio in control (Group A), HFD (Group B) and HFD + BTE (Group C) supplemented groups of rats.

Parameters	Control	High-fat	High-fat + BTE	Significance level^##^	Significance level*	
	(Group A)	(Group B)	(Group C)		Gr. A versus Gr. B	Gr. B versus Gr. C
Urinary calcium (mg/24 h)	0.81 ± 0.24	3.21 ± 0.56	1.01 ± 0.11	*P* < .01	*P* < .01	*P* < .01
Urinary phosphate (mg/24 h)	1.13 ± 0.084	3.21 ± 0.085	1.32 ± 0.206	*P* < .01	*P* < .01	*P* < .01
Urinary creatinine (mg/24 h)	0.45 ± 0.16	0.97 ± 0.021	0.65 ± 0.09	*P* < .01	*P* < .01	*P* < .01
Ca : Cr	1.132 ± 0.16	3.37 ± 0.54	1.48 ± 0.14	*P* < .01	*P* < .01	*P* < .01

Values are expressed as mean ± S.E.M. (*n* = 6). ^##^Denotes significance level based on Kruskal-Wallis nonparametric ANOVA test and * denotes significance level based on Mann-Whitney “U” multiple comparison test.

**Table 2:** Serum alkaline phosphatase (AP) activity and serum tartrate-resistant acid phosphatase (TRAP) activity in control (Group A), HFD (Group B) and HFD + BTE (Group C) supplemented groups of rats.

Serum enzyme activity	Control	High-fat	High-fat + BTE	Significance level^##^	Significance level*	
	(Group A)	(Group B)	(Group C)		Gr. A versus Gr. B	Gr. B versus Gr. C
Alkaline phosphatase (U/L)	9.41 ± 0.22	16.48 ± 3.2	9.97 ± 0.24	*P* < .01	*P* < .01	*P* < .01
TRAP (U/L)	2.1 ± 0.32	4.29 ± 0.26	2.04 ± 0.28	*P* < .01	*P* < .01	*P* < .01

Values are expressed as mean ± S.E.M. (*n* = 6). ^##^Denotes significance level based on Kruskal-Wallis nonparametric ANOVA test and * denotes significance level based on Mann-Whitney “U” multiple comparison test.

**Table 3:** Density of femur, eighth thoracic rib, eighth thoracic vertebra, and fourth lumbar vertebra in control group (Group A), HFD group (Group B), and HFD + BTE supplemented group (Group C) of rats.

Parameters	Control	High-fat	High-fat + BTE	Significance level^##^	Significance level*	
	(Group A)	(Group B)	(Group C)		Gr. A versus Gr. B	Gr. B versus Gr. C
Bone density (gm/cm^3^)						
Right femur	1.39 ± 0.02	1.22 ± 0.01	1.34 ± 0.007	*P* < .01	*P* < .01	*P* < .01
Eighth Thoracic Rib	2.57 ± 0.28	1.44 ± 0.03	2.15 ± 0.11	*P* < .01	*P* < .01	*P* < .01
Eighth Thoracic Vertebra	1.33 ± 0.03	1.23 ± 0.02	1.299 ± 0.01	*P* < .05	*P* < .05	*P* < .01
Fourth Lumbar Vertebra	1.272 ± 0.28	1.44 ± 0.03	2.15 ± 0.11	*P* < .01	*P* < .01	*P* < .01

Values are expressed as mean ± S.E.M. (*n* = 6). ^##^Denotes significance level based on Kruskal-Wallis nonparametric ANOVA test and * denotes significance level based on Mann-Whitney “U” multiple comparison test.

**Table 4:** Bone mineral content of femur, eighth thoracic rib, eighth thoracic vertebra and fourth lumbar vertebra in control group (Group A), HFD group (Group B), and HFD + BTE supplemented groups (Group C) of rats.

Parameters	Control	High-fat	High-fat + BTE	Significance level^##^	Significance level*	
	(Group A)	(Group B)	(Group C)		Gr. A versus Gr. B	Gr. B versus Gr. C
Bone calcium (% of ash weight)						
Femur	19.39 ± 1.09	9.73 ± 0.53	13.75 ± 0.64	*P* < .001	*P* < .01	*P* < .01
Eighth thoracic rib	23.39 ± 0.84	15.49 ± 0.92	23.01 ± 1.38	*P* < .01	*P* < .01	*P* < .01
Eighth thoracic vertebra	24.3 ± 1.39	13.7 ± 0.7	21.52 ± 1.02	*P* < .01	*P* < .01	*P* < .01
Fourth lumbar vertebra	25.68 ± 0.57	13.65 ± 0.78	18.56 ± 0.8	*P* < .001	*P* < .01	*P* < .01

Bone phosphate (% of ash Weight)						
Femur	8.6 ± 0.49	3.88 ± 0.46	7.81 ± 0.62	*P* < .01	*P* < .01	*P* < .01
Eighth thoracic rib	11.89 ± 0.11	6.24 ± 0.45	11.67 ± 0.16	*P* < .01	*P* < .01	*P* < .01
Eighth thoracic vertebra	10.67 ± 0.36	7.21 ± 0.23	10.72 ± 0.43	*P* < .01	*P* < .01	*P* < .01
Fourth lumbar vertebra	11.38 ± 0.39	5.34 ± 0.28	11.56 ± 0.12	*P* < .01	*P* < .01	*P* < .01

Values are expressed as mean ± S.E.M (*n* = 6). ^##^denotes significance level based on Kruskal-Wallis nonparametric ANOVA test and * denotes significance level based on Mann-Whitney “U” multiple comparison test.

## References

[1] Oldenburg B, Pijli H (2001). Abdominal obesity: metabolic complications and consequences for the liver. *Ned Tijdschr Geneeskd*.

[2] Scheen AJ, Luyckx FH (2002). Obesity and liver disease. *Best Practice and Research: Clinical Endocrinology and Metabolism*.

[3] Farrell GC (2003). Non-alcoholic steatohepatitis: what is it, and why is it important in the Asia-Pacific region?. *Journal of Gastroenterology and Hepatology*.

[4] Gonzalez-Calvin JL, Gallego-Rojo F, Fernandez-Perez R, Casado-Caballero F, Ruiz-Escolano E, Olivares EG (2004). Osteoporosis, mineral metabolism, and serum soluble tumor necrosis factor receptor p55 in viral cirrhosis. *Journal of Clinical Endocrinology and Metabolism*.

[5] Gallego-Rojo FJ, Gonzalez-Calvin JL, Muñoz-Torres M, Mundi JL, Fernandez-Perez R, Rodrigo-Moreno D (1998). Bone mineral density, serum insulin-like growth factor I, and bone turnover markers in viral cirrhosis. *Hepatology*.

[6] Weinreb M, Pollak RD, Ackerman Z (2004). Experimental cholestatic liver disease through bile-duct ligation in rats results in skeletal fragility and impaired osteoblastogenesis. *Journal of Hepatology*.

[7] Suzuki K, Takahashi J, Takada H, Kuwayama H (2004). Osteoporosis associated with chronic liver disease. *Nippon Rinsho*.

[8] Van Daele PL, Pols HA (2000). Disorders of bone metabolism in gastrointestinal and hepatic diseases. *Ned Tijdschr Geneeskd*.

[9] Crosbie OM, Freaney R, McKenna MJ, Hegarty JE (1999). Bone density, vitamin D status, and disordered bone remodeling in end-stage chronic liver disease. *Calcified Tissue International*.

[10] Sanchez AJ, Jaime AM (2006). Liver disease and osteoporosis. *Nutrition in Clinical Practice*.

[11] Laroche M, Moulinier L, Bon E, Cantagrel A, Mazieres B (1994). Renal tubular disorders and arteriopathy of the lower limbs: risk factors for osteoporosis in men?. *Osteoporosis International*.

[12] Pinals RS, Jabbs JM (1972). Type-IV hyperlipoproteinaemia and transient osteoporosis. *The Lancet*.

[13] Broulik PD, Kapitola J (1993). Interrelations between body weight, cigarette smoking and spine mineral density in osteoporotic Czech women. *Endocrine Regulations*.

[14] Jie KSG, Bots ML, Vermeer C, Witteman JCM, Grobbee DE (1996). Vitamin K status and bone mass in women with and without aortic atherosclerosis: a population-based study. *Calcified Tissue International*.

[15] Barengolts EI, Berman M, Kukreja SC, Kouznetsova T, Lin C, Chomka EV (1998). Osteoporosis and coronary atherosclerosis in asymptomatic postmenopausal women. *Calcified Tissue International*.

[16] Ouchi Y, Akishita M, De Souza AC, Nakamura T, Orimo H (1993). Age-related loss of bone mass and aortic/aortic valve calcification reevaluation of recommended dietary allowance of calcium in the elderly. *Annals of the New York Academy of Sciences*.

[17] Parhami F, Tintut Y, Beamer WG, Gharavi N, Goodman W, Demer LL (2001). Atherogenic high-fat diet reduces bone mineralization in mice. *Journal of Bone and Mineral Research*.

[18] Xu H, Watkins BA, Seifert MF (1995). Vitamin E stimulates trabecular bone formation and alters epiphyseal cartilage morphometry. *Calcified Tissue International*.

[19] Wohi GR, Loehrke L, Watkins BA, Zernicke RF (1998). Effects of high-fat diet on mature bone mineral content, structure, and mechanical properties. *Calcified Tissue International*.

[20] Krishnamoorthy  KK The nutritional and therapeutic value of tea.

[21] Dufresne CJ, Farnworth ER (2001). A review of latest research findings on the health promotion properties of tea. *Journal of Nutritional Biochemistry*.

[22] Frei B, Higdon JV (2003). Antioxidant activity of tea polyphenols in vivo: evidence from animal studies. *Journal of Nutrition*.

[23] Nag Chaudhuri AK, Karmakar S, Roy D, Pal S, Pal M, Sen T (2005). Anti-inflammatory activity of Indian black tea (Sikkim variety). *Pharmacological Research*.

[24] Vinson JA, Dabbagh YA (1988). Effect of green and black tea supplementation on lipids, lipid oxidation and fibrogen in the hamster: mechanism for the epidemiological benefits of tea drinking. *The FEBS Letters*.

[25] Imai K, Nakachi K (1995). Cross sectional study of effects of drinking green tea on cardiovascular and liver diseases. *British Medical Journal*.

[26] Hegarty VM, May HM, Khaw KT (2000). Tea drinking and bone mineral density in older women. *American Journal of Clinical Nutrition*.

[27] Wu CH, Yang YIC, Yao WJ, Lu FH, Wu JS, Chang CJ (2002). Epidemiological evidence of increased bone mineral density in habitual tea drinkers. *Archives of Internal Medicine*.

[28] Chen Z, Pettinger MB, Ritenbaugh C (2003). Habitual tea consumption and risk of osteoporosis: a prospective study in the women’s health initiative observational cohort. *American Journal of Epidemiology*.

[29] Miksicek RJ (1993). Commonly occurring plant flavonoids have estrogenic activity. *Molecular Pharmacology*.

[30] Uesugi T, Fukui Y, Yamori Y (2002). Beneficial effects of soybean isoflavone supplementation on bone metabolism and serum lipids in postmenopausal Japanese women: a four-week study. *Journal of the American College of Nutrition*.

[31] Mori M, Aizawa T, Tokoro M, Miki T, Yamori Y (2004). Soy isoflavone tablets reduce osteoporosis risk factors and obesity in middle-aged Japanese women. *Clinical and Experimental Pharmacology &amp; Physiology*.

[32] Kanis J, Johnell O, Gullberg B (1999). Risk factors for hip fracture in men from southern Europe: the MEDOS study. Mediterranean Osteoporosis study. *Osteoporosis International*.

[33] Johnell O, Gullberg B, Kanis JA (1995). Risk for hip fracture in European women: the MEDOS study. Mediterranean Osteoporosis Study. *Journal of Bone and Mineral Research*.

[34] Das AS, Mukherjee M, Das D, Mitra C (2009). Protective action of aqueous black tea (Camellia sinensis) extract (BTE) against ovariectomy-induced oxidative stress of mononuclear cells and its associated progression of bone loss. *Phytotherapy Research*.

[35] Das AS, Das D, Mukherjee M, Mukherjee S, Mitra C (2005). Phytoestrogenic effects of black tea extract *(Camellia sinensis)* in an oophorectomized rat *(Rattus norvegicus)* model of osteoporosis. *Life Sciences*.

[36] Das AS, Mukherjee M, Mitra C (2004). Evidence for a prospective anti-osteoporosis effect of black tea *(Camellia Sinensis)* extract in a bilaterally ovariectomized rat model. *Asia Pacific Journal of Clinical Nutrition*.

[37] Chanda S, Islam MN, Pramanik P, Mitra C (1996). High-lipid diet intake is a possible predisposing factor in the development of hypogonadal osteoporosis. *Japanese Journal of Physiology*.

[38] Baumgardner J, Shankar K, Badger TM, Ronis MJ A new rat model for non-alcoholic steatohepatitis.

[39] Dunlap FG, White PJ, Pollak LM (1995). Fatty acid composition of oil from exotic corn breeding materials. *Journal of the American Oil Chemists’ Society*.

[40] Wei H, Zhang X, Zhao JF, Wang ZY, Bickers D, Lebwohl M (1999). Scavenging of hydrogen peroxide and inhibition of ultraviolet light-induced oxidative DNA damage by aqueous extracts from green and black teas. *Free Radical Biology and Medicine*.

[41] Nath RL, Nath RK (1990). Techniques in the biochemical investigation of diseases. *Practical Biochemistry in Clinical Medicine*.

[42] Adeniyi KO, Ogunkeye OO, Isichei CO (1993). Thryroidectomy and thyroxine administration alter serum calcium levels in rat. *Acta Physiologica Hungarica*.

[43] Lowry HO, Lopez AJ (1946). The determination of inorganic phosphate in the presence of labile phosphate esters. *The Journal of Biological Chemistry*.

[44] Nath RL, Nath RK (1990). Tests for renal function-investigation of nephrotic syndrome and other primary organ related disorders. *Practical Biochemistry in Clinical Medicine*.

[45] Michell RH, Karnovsky MJ, Karnovsky ML (1970). The distributions of some granule-associated enzymes in guinea-pig polymorphonuclear leucocytes. *Biochemical Journal*.

[46] Abbott L, Kaplan A (1984). Acid phosphatase. *Clinical Chemistry*.

[47] Arjmandi BH, Alekel L, Hollis BW (1996). Dietary soybean protein prevents bone loss in an ovariectomized rat model of osteoporosis. *Journal of Nutrition*.

[48] Shapiro R, Heaney RP (2003). Co-dependence of calcium and phosphorus for growth and bone development under conditions of varying deficiency. *Bone*.

[49] Yeh JK, Liu CC, Aloia JF (1993). Effects of exercise and immobilization on bone formation and resorption in young rats. *American Journal of Physiology: Endocrinology and Metabolism*.

[50] Islam MN, Chanda S, Mitra C (2000). Effects of different intensities of cold stress on certain physiological phenomena related to skeletal health in a hypogonadal rat model. *Journal of physiology and pharmacology*.

[51] Maenz DD, Cheeseman CI (1986). Effect of hyperglycemia on D-glucose transport across the brush-border and basolateral membrane of rat small intestine. *Biochimica et Biophysica Acta*.

[52] Koyama I, Tsugikazu K, Yoshikatsu S, Munetsugu K (1983). A possible mechanism for the changes in hepatic and intestinal alkaline phosphatase activities in bile duct ligated rats and guinea pigs. *Biochimica et Biophysica Acta*.

[53] Lowry OH, Rosenbrough NJ, Farr AL, Randall RJ (1951). Protein measurement with the folin phenol reagent. *The Journal of Biological Chemistry*.

[54] Rorive G, Kleinzeller A (1974). Ca^2+^ activated ATPase from renal tubular cells. *Methods in Enzymology*.

[55] Miao D, Scutt A (2002). Recruitment, augmentation and apoptosis of rat osteoclasts in 1, 25-(OH)_2_D_3_ response to short-term treatment with 1,25-dihydroxyvitamin D_3_
*in vivo*. *BMC Musculoskeletal Disorders*.

[56] Mahdy KA, Ahmed HH, Mannaa F, Abdel-Shaheed A (2007). Clinical benefits of biochemical markers of bone turnover in Egyptian children with chronic liver diseases. *World Journal of Gastroenterology*.

[57] Schiefke I, Fach A, Wiedmann M (2005). Reduced bone mineral density and altered bone turnover markers in patients with non-cirrhotic chronic hepatitis B or C infection. *World Journal of Gastroenterology*.

[58] Gharavi N (2002). Role of lipids in osteoporotic bone loss. *Nutrition Bytes*.

[59] Xiao XX, Hara I, Matsumiya T (2002). Effects of osthole on postmenopausal osteoporosis using ovariectomized rats: comparison to the effects of estradiol. *Biological and Pharmaceutical Bulletin*.

[60] Lindsay R, Coutts JRT, Hart DM (1977). The effect of endogenous oestrogen on plasma and urinary calcium and phosphate in oophorectomized women. *Clinical Endocrinology*.

[61] Myburgh KH, Noakes TD, Roodt M, Hough FS (1989). Effect of exercise on the development of osteoporosis in adult rats. *Journal of Applied Physiology*.

[62] Delmas PD (1993). Biochemical markers of bone turnover. *Journal of Bone and Mineral Research*.

[63] Gertz BJ, Shao P, Hanson DA (1994). Monitoring bone resorption in early postmenopausal women by an immunoassay for cross-linked collagen peptides in urine. *Journal of Bone and Mineral Research*.

[64] Stepan  JJ, Moss  DW, Rosalki  SB (1996). Enzyme tests in bone disease. *Enzyme Tests in Diagnosis*.

[65] Jiang YN, Mo HY, Chen JM (2002). Effects of epimedium total flavonoids phytosomes on preventing and treating bone-loss of ovariectomized rats. *Zhongguo Zhong yao Zazhi*.

[66] Li B, Yu S (2003). Genistein prevents bone resorption diseases by inhibiting bone resorption and stimulating bone formation. *Biological and Pharmaceutical Bulletin*.

[67] Simpson ER, Davis SR (2001). Minireview: aromatase and the regulation of estrogen biosynthesis: some new perspectives. *Endocrinology*.

[68] Simpson ER, Clyne C, Speed C, Rubin G, Bulun S (2001). Tissue-specific estrogen biosynthesis and metabolism. *Annals of the New York Academy of Sciences*.

[69] Chung H, Kang YS, Hwang CS (2001). Deflazacort increases osteoclast formation in mouse bone marrow culture and the ratio of RANKL/OPG mRNA expression in marrow stromal cells. *Journal of Korean Medical Science*.

[70] Wills MR (1973). Intestinal absorption of calcium. *The Lancet*.

[71] Gennari C, Imbimbo B, Montagnani M, Bernini M, Nardi P, Avioli LV (1984). Effects of prednisole and deflazacort on mineral metabolism and parathyroid hormone activity in humans. *Calcified Tissue International*.

[72] Chanda S, Islam N, Ghosh TK, Mitra C (1999). Effects of a high intake of unsaturated and saturated oils on intestinal transference of calcium and calcium mobilization from bone in an ovariectomized rat model of osteoporosis. *Asia Pacific Journal of Clinical Nutrition*.

[73] Ghijsen  WEJM, DeJong MD, Van Os CH (1980). Association between Ca^2+^ ATPase and alkaline phosphatase activities in plasma membranes of rat duodenum. *Biochimica et Biophysica Acta*.

[74] Bolscher MT, Netelenbos JC, Barto R, Van Buuren LM, Van Der Vijgh WJF (1999). Estrogen regulation of intestinal calcium absorption in the intact and ovariectomized adult rat. *Journal of Bone and Mineral Research*.

[75] Cotter AA, Jewell C, Cashman KD (2003). The effect of oestrogen and dietary phyto-oestrogens on transepithelial calcium transport in human intestinal-like Caco-2 cells. *British Journal of Nutrition*.

[76] Arjmandi BH, Hollis BW, Kalu DN (1994). In vivo effect of 17*β*-estradiol on intestinal calcium absorption in rats. *Bone and Mineral*.

[77] Salih MA, Sims SH, Kalu DN (1996). Putative intestinal estrogen receptor: evidence for regional differences. *Molecular and Cellular Endocrinology*.

